# Editorial: “Linking Hypoxia to Obesity”

**DOI:** 10.3389/fendo.2017.00034

**Published:** 2017-02-22

**Authors:** Daniela P. Foti, Antonio Brunetti

**Affiliations:** ^1^Department of Health Sciences, University “Magna Graecia” of Catanzaro, Catanzaro, Italy

**Keywords:** hypoxia, HIF-1, HIF-2, obesity, adipose tissue

**The Editorial on the Research Topic**

**Linking Hypoxia to Obesity**

Obesity is a major public health problem that constitutes a worldwide epidemic, often associated with metabolic and cardiovascular diseases, and some types of cancers (1). The rising prevalence of obesity and obesity-related medical conditions has therefore promoted much interest and research on the role of adipose tissue, previously seen simply as an inert lipid storage site. In the last two decades, many biologically active molecules produced by fat have been identified, evoking a role of this tissue in previously unsuspected processes, including inflammation, insulin, and glucose homeostasis, angiogenesis, and hemostasis (2). Hormonal factors produced by adipose cells have been collectively called “adipokines”, and include about a hundred of known molecules, such as leptin, adiponectin, resistin, apelin, visfatin, VEGF, PAI-1, and many other, not yet identified factors, that have recently emerged by proteomic strategies (3, 4). It has been postulated that the adipose tissue of the obese, becoming dysfunctional by changes in the secretion pattern of adipokines, may sustain inflammation, insulin resistance, and a pro-thrombotic state with endothelial dysfunction, all conditions underpinning obesity-related comorbidities (5, 6). The notion of obesity as a systemic, low-grade inflammatory state promoting insulin resistance has become commonly accepted (7), although the event(s) and the related molecular mechanisms that initially trigger adipose tissue dysfunction have remained still poorly characterized. In this regard, a direct role of hypoxia in adipose and non-adipose cells in fat tissue has been demonstrated both *in vivo*, in animal models, and in *in vitro* studies (8–11). Oxidative stress, endoplasmic reticulum stress, and the activation of the unfolded protein response can represent additional cellular processes, indirectly linked to hypoxia, which may operate in hypertrophic fat cells.

The aim of this research topic was to provide insights into the mechanisms triggered by hypoxia in obesity.

Hypoxia-inducible factor 1 (HIF-1) is considered the main nuclear transcription factor that mediates cellular response to low oxygen tension (12). Although the scenario is far from be complete, many HIF-1-inducible genes have been identified in the adipose tissue, including *leptin, VEGF, GLUT1*, metalloproteinases *MMP2* and *MMP9, IL-4, IL-6*, and *PAI-1*, thereby implicating a role of hypoxia in important processes, such as inflammation, insulin resistance and glucose intolerance, and angiogenesis (9, 13, 14). In this context, the molecular interplay between HIF-1 and other nuclear partners involved in the gene networks affected by oxygen availability (i.e., NF-κB, HIF-2, CREB, PPARγ) deserves further investigation.

Messineo et al. contributed a research article on the physical and functional interaction between HIF-1 and HMGA1, two factors that share important roles in adipose tissue and inflammation. Using *in vitro* differentiated 3T3-L1 adipocytes, cooperation between HIF-1 and HMGA1 proteins was demonstrated for the first time in the transcriptional activation of the hypoxia-responsive *Vegf* and *visfatin* genes, suggesting that HMGA1 may represent a novel nuclear partner of HIF-1, thereby serving as a modulator of HIF-1 activity in hypoxia. A more detailed analysis of the molecular cross-talk between these two factors may help identifying protein and gene networks relevant to obesity.

A plethora of studies using transgenic models have addressed the role of the HIFs on adipose dysfunction (15–18). Although findings are complex to interpret and sometimes contradictory, evidences exist that the HIF pathway does contribute to the obese phenotypes.

Lin and Yun reviewed the role of HIF-2 in hypertrophic cardiomyopathy, a condition that may occur in obesity independently of other cardiovascular risk factors, including hypertension, diabetes, and coronary heart disease. The Authors drew attention to the fact that stabilization of HIF-2, as obtained in genetically engineered mice, is followed by fat inflammation and lethal cardiomegaly, pointing to a novel pathophysiological role of HIF-2 in driving NF-κB and NFAT activation, that may ultimately compromise heart development, morphology, and function.

Goossens and Blaak focused into the controversial relevance of oxygen in human obesity and delineated future directions. Starting from the consolidated notion that adipose tissue hypoxia plays an important role in adipose dysfunction in mouse models of obesity, the Authors summarized current findings in human obesity, underlining how methodological differences may account for discrepant results (i.e., measurements of adipose tissue oxygen partial pressure and metabolic markers). Accordingly, advancements are expected from studies in progress with obese patients before and after weight loss and from techniques that allow for a better characterization of the degree, severity, and pattern of hypoxia.

Heinonen et al. reviewed the effects of hypoxia and the consequent physiological adaptation to altitude exposure and physical exercise, in terms of cardiovascular and metabolic responses. While focusing on conditions implicating hypoxia in adipose tissue during rest and moderate or intense exercise, the Authors discussed the mechanisms that influence adipose tissue blood flow in each condition and considered the positive and negative consequences of hypoxia and hyperoxia in relation to treatment of obesity.

The effects of hypoxia on the different adipose tissue depots (white, brown, and brite) were discussed in a paper by Trayhurn and Alomar, where Authors focused also on the less explored brown adipose tissue, in which reduced oxygen tension in obese mice leads to whitening of brown fat and mitochondrial loss and dysfunction. They hypothesized that, as in white adipose tissue, oxygen deprivation in brown fat may trigger changes in adipokine secretion, increase glucose uptake and lactate release, reduce free fatty acid uptake, and promote fibrosis and insulin resistance, thus contributing to the development of the metabolic syndrome. In an emerging complex scenario, as a consequence of obesity and hypoxia, the production of lactate by anaerobic metabolism in the white adipose tissue (19), through the stimulation of UCP1, promotes thermogenesis *via* lipid oxidation and the development of brite cells within white depots, leading to browning of white fat.

In summary, these articles have contributed importantly to the current debate linking hypoxia to obesity. Our hope is that a better understanding of the role of hypoxia in adipose tissue will contribute to deepen our knowledge on the pathophysiology of obesity and obesity-related disorders, leading to innovative targets for preventive and therapeutic interventions.

## Author Contributions

All authors listed have made substantial, direct, and intellectual contribution to the work and approved it for publication.

## Conflict of Interest Statement

The authors declare that the research was conducted in the absence of any commercial or financial relationships that could be construed as a potential conflict of interest.
